# Effects of cold seawater pre-treatments on induction of early sexual maturation and sperm production in European eel (*Anguilla anguilla)*

**DOI:** 10.1007/s10695-024-01402-w

**Published:** 2024-09-05

**Authors:** L. Ferrão, M. Morini, W. A. González-Lopéz, V. Gallego, A. Felip, L. Pérez, J. F. Asturiano

**Affiliations:** 1 Grupo de Acuicultura y Biodiversidad, Instituto de Ciencia y Tecnología Animal, Universitat Politècnica de València. Camino de Vera S/N, 46022 Valencia, Spain; 2 Department of Fish Physiology and Biotechnology, Instituto de Acuicultura de Torre de La Sal (IATS), CSIC, 12595 Ribera de Cabanes, Castellón Spain

**Keywords:** *Anguilla anguilla*, Maturation, Testis, Spermatogonia, Temperature

## Abstract

To induce sexual maturation in captivity, eels rely on hormonal treatments, but this process is costly and time-consuming. As an alternative, different types of conditioning, also referred as pre-treatment, have been assessed to ease hormonal treatment response. Recent studies have shown that migrating eels experience a wide range of temperatures, varying from 12 °C at night to as low as to 8 °C during the day. Therefore, this study evaluates the effects of low-temperature (10 °C) seawater pre-treatments of different durations (2 and 4 weeks) on male eel reproduction. The eye, gonadosomatic and hepatosomatic indexes from control (without thermic seawater pre-treatment) and pre-treated fish were measured. Blood and testis samples were also collected for sex steroid and histology analysis, respectively. Eels pre-treated for 2 weeks demonstrated increased progestin levels, comparing with the control group. Eels pre-treated for 4 weeks showed significantly higher gonadosomatic index and elevated androgens and estradiol levels in comparison with the remaining groups. In eels pre-treated for 2 and 4 weeks, there was an increase in the proportion of spermatogonia type B cells compared to undifferentiated spermatogonia type A, a differentiation process that was not observed in the control group. Cold seawater pre-treatment induced early sexual maturation, including steroid production, which consequently stimulated biometric changes and increased spermatogonia differentiation. Following the pre-treatments, eels started receiving standard hormonal treatment (with recombinant human chorionic gonadotropin at 20 °C). Pre-treated males started to spermiate earlier than the control group. In some treatment weeks, pre-treated individuals registered higher values of sperm density, motility, and kinetic parameters. Moreover, an economic evaluation was carried out relating the investment made in terms of hormone injections with the volume of high-quality sperm obtained from each experimental group. The low temperature pre-treatments demonstrated their economic effectiveness in terms of hormone treatment profitability, increasing the production of high-quality sperm in the European eel. Thus, this in vivo study suggests that cold seawater pre-treatment may increase sensitivity to the hormone applied during standard maturation treatment.

## Introduction

The European eel (*Anguilla anguilla*) is a catadromous fish with a complex life cycle, including various morphological and physiological changes prior to a long transatlantic reproductive migration. It is considered a prized product in the European and Asian markets, making it a target for fisheries and aquaculture, which until now has been based on catching juveniles and growing them until commercial size. However, overfishing of juvenile glass eels or during their adult phase, together with other factors such as pollution, the construction of dams, viruses, parasites, etc., has led to a drastic decline in the population of the species, which has been included on the Red List of the International Union for Conservation of Nature (IUCN) as ‘critically endangered’ (Pike et al. 2020). Therefore, reproduction in captivity conditions for both the aquaculture industry and potential restocking programs is considered a necessity to reduce the pressure on natural populations while supplying glass eels to farms.

Nowadays, it is possible to obtain both eggs and sperm of this species to perform in vitro fertilizations after using hormonal treatments (Butts et al. 2014; Di Biase et al. 2016; Herranz-Jusdado et al. 2019a; Jéhannet et al. 2024). However, this approach still presents various limitations, specifically its high cost and the extended duration of hormonal treatments required for fish maturation (several weeks in males and various months in females; Pérez et al. 2000; Gallego et al. 2012). Moreover, in the Japanese eel, although the life cycle has been successfully closed, low egg quality and fertilization rates still represent a major problem in sustainable eel aquaculture (Burgerhout et al. 2019). It has been suggested that the low survival rates during embryo and larvae stage in teleosts may be caused by the low quality of gametes, partly due to uncontrolled epigenetic factors (Herráez et al. 2017). Another factor that might negatively affect the reproduction success in eels is the lack of natural triggers (e.g. temperature, salinity, photoperiod) in captivity conditions (Burgerhout et al. 2019). Thus, there is a need to assess the effect of manipulating natural triggers, which could disclose more efficient alternatives to hormonal treatments. This approach may ensure high-quality gametes while maximizing the profits in the eel aquaculture practice.

Like other teleost species, silvering eels need to remove the dopaminergic inhibition to gonadotrophins production, that stimulate gonadal development (Vidal et al. 2004). In wild migrating silver eels, this removal process seems to be triggered by changes in the environmental conditions (Dufour et al. 2003; Sébert et al. 2008). The term ‘pre-treatments’ (often known as ‘conditioning’) refers to the use of environmental or hormonal factors that aim to induce the first phases of the maturation process and the gonadal development or, at least, to increase the sensitivity of the eels to the hormonal treatments applied consecutively. The application of these pre-treatments can be especially important for maturing farmed eels, as their captivity prevents them from experiencing the natural environmental changes that occur during several phases of the reproductive cycle (Palstra and van den Thillart 2009). Several factors, including temperature, salinity, forced swimming, administration of androgens and even broodstock feeding, have been considered for pre-treatment (Mazzeo et al. 2014; Lokman et al. 2015; Mes et al. 2016; Di Biase et al. 2017; Palstra et al. 2018; Rozenfeld et al. 2019).

Indeed, the use of environmental factors, along with standard hormonal treatments for male European eel maturation, has become a focus of interest in recent years. Once only early type A spermatogonia are found in the testis of the immature European eel (Peñaranda et al. 2016; Morini et al. 2017a), intraperitoneal administration of hormones for several weeks is required to induce spermatogenesis, spermiogenesis and spermiation. Hormone treatments are usually based on the administration of gonadotropins (Pérez et al. 2000; Gallego et al. 2012; Mylonas et al. 2017) after gradual acclimation to seawater while fish are kept at high temperatures (around 20 °C). These hormonal treatments (usually human chorionic gonadotropin, hCG) produce good results in terms of sperm volume, density, and motility parameters (especially when recombinant hCG, hCG_rec_, is used; Gallego et al. 2012; Herranz-Jusdado et al. 2019a). However, our team aimed to better understand the endocrine mechanisms controlling eel spermatogenesis and to improve sperm production, whilst taking into account both hormonal costs and the quantity of high-quality sperm produced (Peñaranda et al. 2016; Herranz-Jusdado et al. 2018; Rozenfeld et al. 2019). Considering the seawater temperatures to which European eels are exposed during their transatlantic migration (Aarestrup et al. 2009; Palstra and van den Thillart 2009), several experiments have been undertaken. Peñaranda et al. (2016) demonstrated the effect of the temperature on male eel maturation, and how low temperatures can delay spermiogenesis induced hormonally. Indeed, fish reared at 10 °C arrested their maturation at the early stage (with a high proportion of spermatogonia type A cells, SPGA), and no further maturation was observed until the temperature was increased (over a threshold of 15 °C). The increase in temperature caused variations in the gene expression of steroidogenic enzymes and lead to a change in the steroidogenic pathway from androgens (synthetized even at low temperatures, linked to the gene expression of other steroidogenic enzymes) towards the synthesis of estrogen and progestin, enabling the following stages of testis maturation (Peñaranda et al. 2016). Later, our group compared the effects of 2-week pre-treatments with seawater at 10 or 20 °C (without applying any hormonal treatment). Rozenfeld et al. (2019) described how the male eels maintained at 10 °C showed a significantly different transcriptome in the brain-pituitary-gonad (BPG) axis and evidenced a thermic sensitization process with significant gene upregulation following the cold seawater treatment. In addition, testis from eels maintained at 10 °C exhibited spermatogonial cell proliferation and differentiation as a response to the higher plasma levels of T and 11-KT induced by low temperature. These results confirmed that temperature has a clear effect on the sexual maturation in the European eel. Furthermore, these studies suggest that cold seawater could be used as a pre-treatment in an attempt to induce the initial stages of the maturation process or to increase the sensitivity of the eels to the hormonal treatments applied consecutively. This approach of thermic pre-treatment using cold seawater in the European eel has not been assessed previously.

The present study evaluates the effects of low temperature (10 °C) seawater pre-treatments of two durations (2 and 4 weeks) during early spermatogenesis and prior to the application of the standard hormonal treatment to induce the sexual maturation in the European eel (weekly administration of hCG_rec_ in seawater at 20 °C). Their effects on the biometric parameters, the spermatogonial maturation process, the plasma levels of steroids and further reproductive performance in terms of sperm quantity and quality were evaluated. Additionally, the economic estimation of hormonal treatment profitability in terms of production of high-quality sperm was calculated in males that received the standard hormonal treatment and those that received a previous cold seawater pre-treatment.

## Materials and methods

### Experimental design

Seventy-two male eels (mean body weight = 89.8 ± 17.0 g) maintained in freshwater at the fish farm Valenciana de Acuicultura, S.A. (Puzol, Valencia, Spain) were transferred to the Fish Reproduction Laboratory in the Universitat Politècnica de València (Valencia, Spain). Following their arrival, fish were distributed randomly in 150-L tanks (12 fish/ tank; 2 tanks/ treatment) filled with freshwater (approximately at 20 °C) with similar conditions to those used in the farm and equipped with separate recirculation systems and temperature control systems (with heaters and coolers). The tanks were covered to reduce light intensity and to reduce fish stress.

**Fig. 1:**
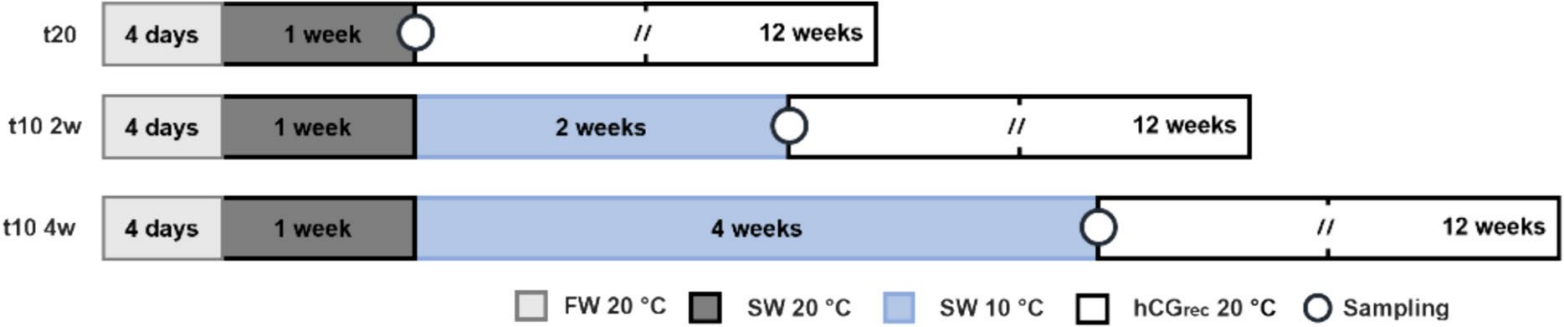
Representation of experimental design. Fish were maintained in freshwater (approximately at 20 °C for 4 days (FW 20 °C). Following this period, eels were acclimatized to seawater at 20 °C (SW 20 °C) for 7 days and after this period, control eels (t20) were sampled and began receiving weekly standard hormonal treatment (hCGrec 20 °C) for 12 weeks. The remaining eels were divided into two groups, that were maintained in cold seawater at 10 °C for 2 weeks (t10 2w) and 4 weeks (t10 4w). After pre-treatments, the temperature was increased to 20 °C and pre-treated males started receiving standard hormonal treatment as the control group. Samplings are shown with circles

The fish were maintained in freshwater conditions for 4 days before being gradually acclimatized to seawater (37.0 ± 0.3‰ of salinity) at 20 °C for one week (Fig. 1). Following seawater adaptation, a group of 8 eels were sacrificed and served as control group (t20). The eels from the control group that were not sacrificed started immediately to receive standard hormonal treatment (weekly injections of hCG_rec_; Ovitrelle®, Madrid, Spain, 1.5 IU/g fish) administrated intraperitoneally for 12 weeks. The remaining eels were divided into two groups for the cold seawater pre-treatment, being transferred and maintained in seawater at 10 °C for 2 and 4 weeks (t10 2w and t10 4w). Once each pre-treatment finished, 8 eels per group were sacrificed for samplings. The water temperature from the pre-treated groups was subsequently increased to 20 °C and each group started to receive the standard hormonal treatment for 12 weeks as the control group.

### Samplings

Biometric measurements from sampled fish (n = 8 for each experimental group) were registered and included total weight, total length, vertical and horizontal eye diameters, testis weight and liver weight. From these measurements, the eye index [(100 × π × 0.25 (horizontal distance + vertical distance)^2^ / total length; Pankhurst 1982)], hepatosomatic index [(liver weight / total weight) × 100] and gonadosomatic index [(gonad weight / total weight) × 100] were calculated.

Blood samples were collected using heparinized syringes and centrifuged at 3500 rpm for 15 min at 4 °C to obtain blood plasma that was stored at -80 °C until further steroid analysis. For histological analysis, testis samples were fixed in 4% glutaraldehyde diluted in phosphate buffer (pH adjusted to 7.4).

Once spermiation started in each group, sperm samples were collected 24 h after the weekly administration of hCG_rec_ to obtain the highest quality sperm (as described by Pérez et al. 2000). The genital area was carefully cleaned with distilled water and thoroughly dried with paper to avoid contamination with feces, urine, or seawater. The sperm was collected by applying a gentle abdominal massage from the pectoral fins to the genital area and with the help of a vacuum pump, the sperm was collected in plastic Falcon tubes (as described by Herranz-Jusdado et al. 2019b). Sperm volume was registered (mL/100 g fish), samples were diluted 1:9 (sperm: extender) in P1 medium [in mM: NaCl 125, NaHCO_3_ 20, MgCl_2_ 2.5, CaCl_2_ 1, KCl 30; pH adjusted to 8.5, described by Peñaranda et al. (2010)] and then maintained at 4 °C until further sperm evaluation.

### Blood plasma steroid assays

Blood plasma levels (n = 8 for each experimental group and sampling) of testosterone (T), 11-ketotestosterone (11-KT), 17β-estradiol (E2), and 17α,20β-dihydroxy-4-pregnen-3-one (DHP) were determined by specific enzyme immunoassays (EIA) following the methods developed for European sea bass (Molés et al. 2011; Rodríguez et al. 2000, 2005) and previously validated for eel plasma (Peñaranda et al. 2018). The lower limits of detection (80% binding) were 0.33 ng/mL for E2, 0.016 ng/mL for T, 0.0023 ng/mL for 11-KT and 0.007 ng/mL for DHP. The inter-assay coefficients of variation were 0.95% (n = 8) for E2, 3.45% (n = 7) for T, 1.85% (n = 7) for 11-KT and 2.43% for DHP (n = 7).

### Testis histology

Testis samples (n = 7–8 for each experimental group and sampling) were dehydrated in increasing percentages of ethanol and embedded in resin (Technovit 7100) in accordance with the instructions of the manufacturer. Sections of 5 µm thickness were cut with a Microm HM325 microtome and stained with 1% toluidine blue. The slides were observed with a Nikon Eclipse E-400 microscope, and pictures were taken with a Nikon DS-5 M camera attached to the microscope (Nikon, Tokyo, Japan).

Cell types were categorized as described by Rozenfeld et al. (2019) for the European eel. The undifferentiated spermatogonia type A cells (SPGAund) showed irregular nuclear membranes and were found isolated. Differentiated SPG type A cells (SPGAdiff) formed groups of 2–8 cells within Sertoli cell surroundings, featuring regular nuclear envelopes, one or more nucleolus and a cytoplasm darker than that found in SPGAund. SPG type B cells (SPGB) were smaller cells with small amounts of cytoplasm and nuclei with large amounts of heterochromatin. FIJI/ImageJ software was used to count the number of each cell type from 5 microscope fields per sample within each experimental group. Cell distribution counts were transformed into percentage data (sum of each cell type found in each slide/sum of total cell counts × 100).

### Sperm quantity and quality evaluation

Sperm volume was evaluated by registering the volume using graduated Falcon tubes. For sperm density assessment, a Neubauer Improved haemocytometer chamber was used for counting sperm under a microscope (Nikon Eclipse 55i, Nikon Corporation) at 40 × magnification. The mean of three counts per male was used for statistical analyses and results are expressed as sperm cells × 10^10^ mL.

Sperm quality was evaluated following the method described by Gallego et al. (2013a, b). Each sperm sample was activated by mixing 0.5 µL of sperm previously diluted in P1 medium with 4.5 µL of artificial seawater (ASW; Aqua Medic Meersalz, 37 g/l) with 2% (w:v) bovine serum albumin (BSA), pH adjusted to 8.2 and osmolality of 1100 mOsm/kg. The sperm activation was performed in an ISAS Spermtrack 10 counting chamber (Proiser R + D, S.L., Spain) on a microscope in negative phase with a 10 × magnification (Nikon Eclipse 80i) connected to a computer with an ISAS 782 M camera (Proiser R + D, S.L., Spain), recording 60 frames per second. All samples were analyzed 15 s after activation, using the CASA module ISAS v1 software (Proiser R + D, S.L., Spain). The parameters considered for analysis were total motility (MOT; %), defined as the percentage of motile spermatozoa; progressive motility (pMOT; %), defined as the percentage of spermatozoa swimming in a straight line; curvilinear velocity (VCL; μm/s), defined as the velocity of a sperm head along its curvilinear trajectory; straight-line velocity (VSL; μm/s), defined as the velocity of a sperm head along the straight line between its first detection and its last position; and average path velocity (VAP; µm/s), defined as the velocity along a derived smoothed path.

### Hormonal treatment profitability

To estimate the economic profitability of administering the hormonal injections during standard treatment prior to cold seawater pre-treatment, three factors were considered: i) the hormone price; ii) the total amount of hormone used during the entire standard treatment of each male; and iii) the total volume of sperm with motility equal or higher than 70% of motile cells produced by each male. The aim was to relate the investment made in terms of hormone injection expenses with the level of high-quality sperm obtained from each experimental group during standard treatment.

### Statistical analysis

Data were expressed as means ± standard error and results expressed in percentages were normalized by arcsine transformation. Shapiro–Wilk and Levene tests were used to verify the normality of data distribution and variance homogeneity, respectively.

One-way analysis of variance (ANOVA) was used to compare the eye index, steroid levels, spermatogonia cell type proportions between the control and pre-treated experimental groups, and to compare sperm quantity and quality parameter values each week between the three experimental groups. Significant differences were detected using the Student–Newman–Keuls (SNK) as a *post-hoc* test (*p* < 0.05). Non-parametric Kruskal–Wallis was used for non-normally distributed or not-equal variant data, to compare the gonadosomatic and hepatosomatic indexes between experimental groups and sperm quantity and quality parameters in the same weeks.

All the statistical analyses were performed using IBM SPSS Statistics for Windows software version 25.0 (Armonk, NY, USA).

## Results

### Biometric parameters

**Fig. 2:**
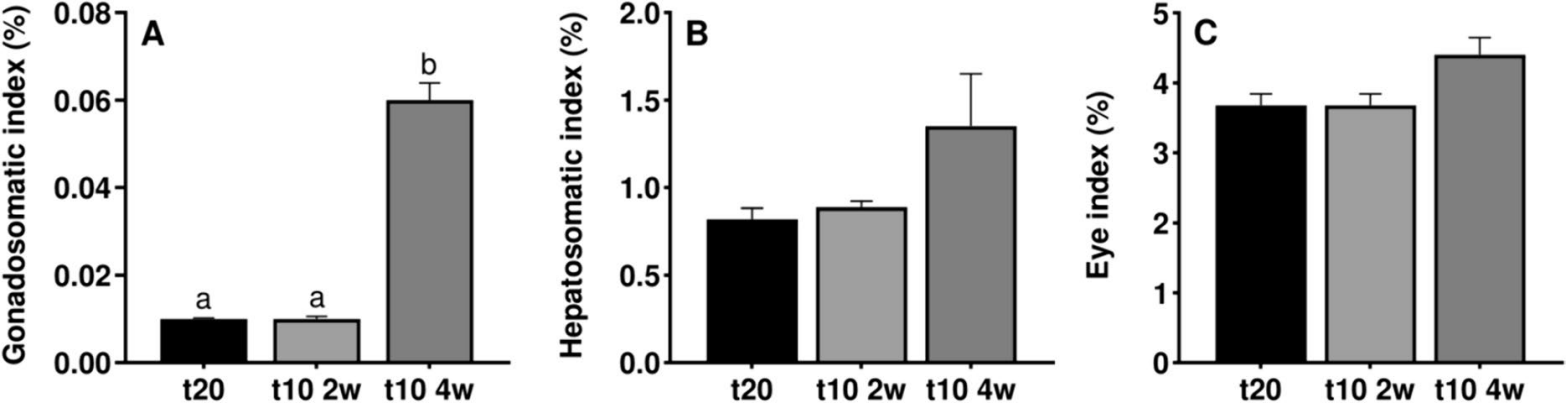
Biometric parameter results. (**A**) gonadosomatic, (**B**) hepatosomatic and (**C**) eye indexes from control (t20), 2-week (t10 2w), and 4-week (t10 4w) pre-treated groups (n = 8 per treatment and sampling). Data are expressed as mean ± SEM and different letters indicate significant differences (one-way ANOVA followed by *pos-hoc* SNK test or Kruskal–Wallis, *p* < 0.05) between treatment in each biometric parameter

Despite all the groups showed low gonadosomatic indexes, the gonadosomatic index of male eels following 4 weeks of cold seawater pre-treatment, registered significantly higher values than those from the control and the 2-week pre-treatment groups (*p* < 0.001) (Fig. 2A). The hepatosomatic (*p* = 0.071) and eye (*p* = 0.123) indexes showing similar increasing patterns, but no significant differences were found (Fig. 2B and C).

### Testis histology

During testis histology analysis, three types of cells were registered (Fig. 3.1 A-C). No differences were found when comparing the percentages of SPGAund (*p* = 0.470), SPGAdiff (*p* = 0.317) and SPGB (*p* = 0.599) cells between the three experimental groups. However, within each experimental group, 2 (*p* < 0.001) and 4-week (*p* < 0.001) pre-treated groups showed increased differentiation of SPGAund into advanced stage cells, namely SPGAdiff and SPGB cells (Fig. 3.2. A-C). Contrary, the control group (*p* < 0.001) showed higher SPGAdiff percentages compared with SPGAund but also with SPGB cells.

**Fig. 3:**
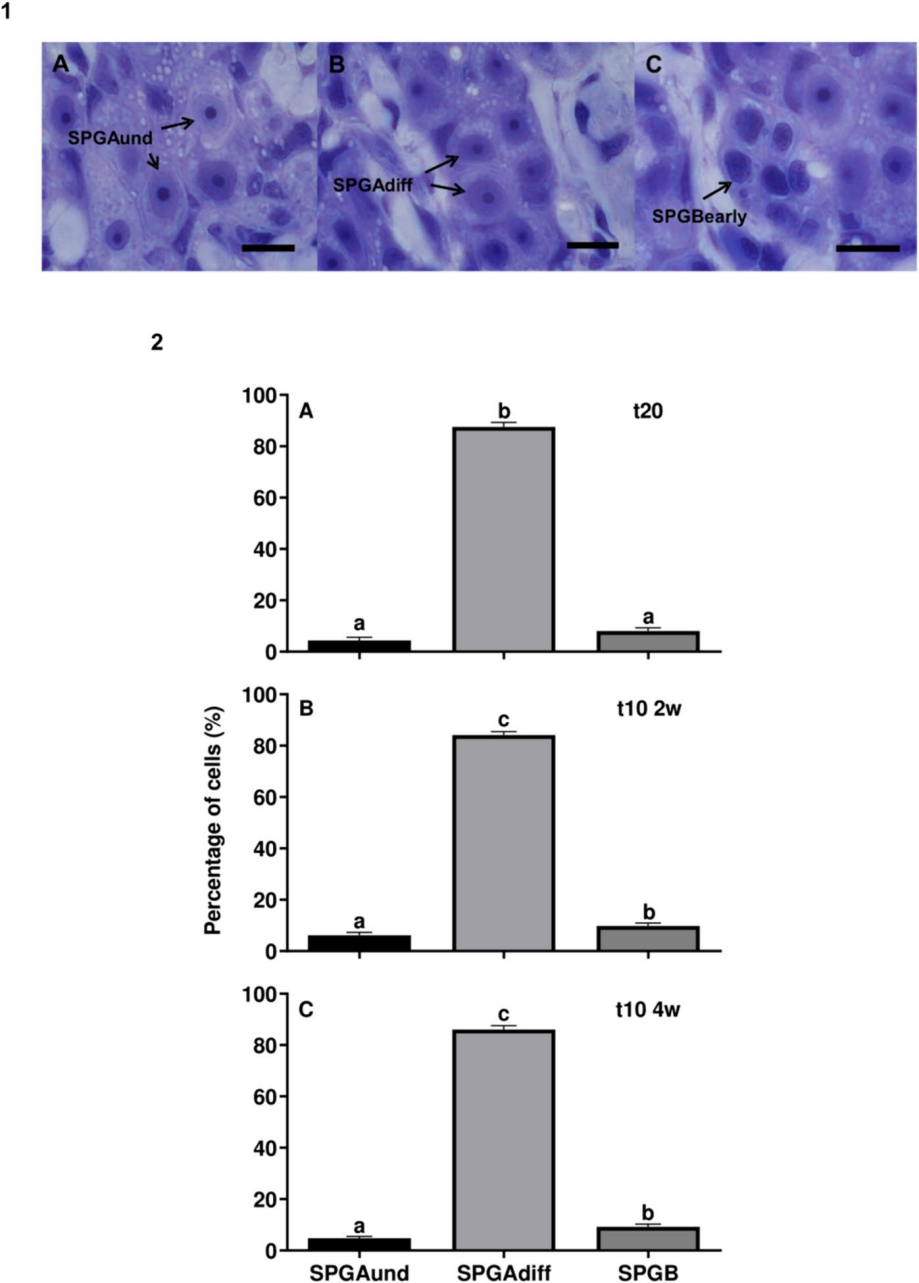
Histology analysis results. (1) Cell types found in the testis samples. (**A**) undifferentiated type A spermatogonia (SPGAund); (**B**) differentiated type A spermatogonia (SPGAdiff) and (**C**) early type B spermatogonia (SPGB). Scale bars = 10 µm. (2) Cell type percentages. Percentages of undifferentiated type A spermatogonia (SPGAund), differentiated type A spermatogonia (SPGAdiff) and early type B spermatogonia (SPGB) in testis from (**A**) control (t20), (**B**) 2-week (t10 2w), and (**C**) 4-week (t10 4w) pre-treated groups (n = 7–8 per treatment and sampling). Data are expressed as mean ± SEM and different letters indicate significant differences (one-way ANOVA followed by *pos-hoc* SNK test, *p* < 0.05) between the mean of cell types in each treatment

### Blood steroid analyses

**Fig. 4:**
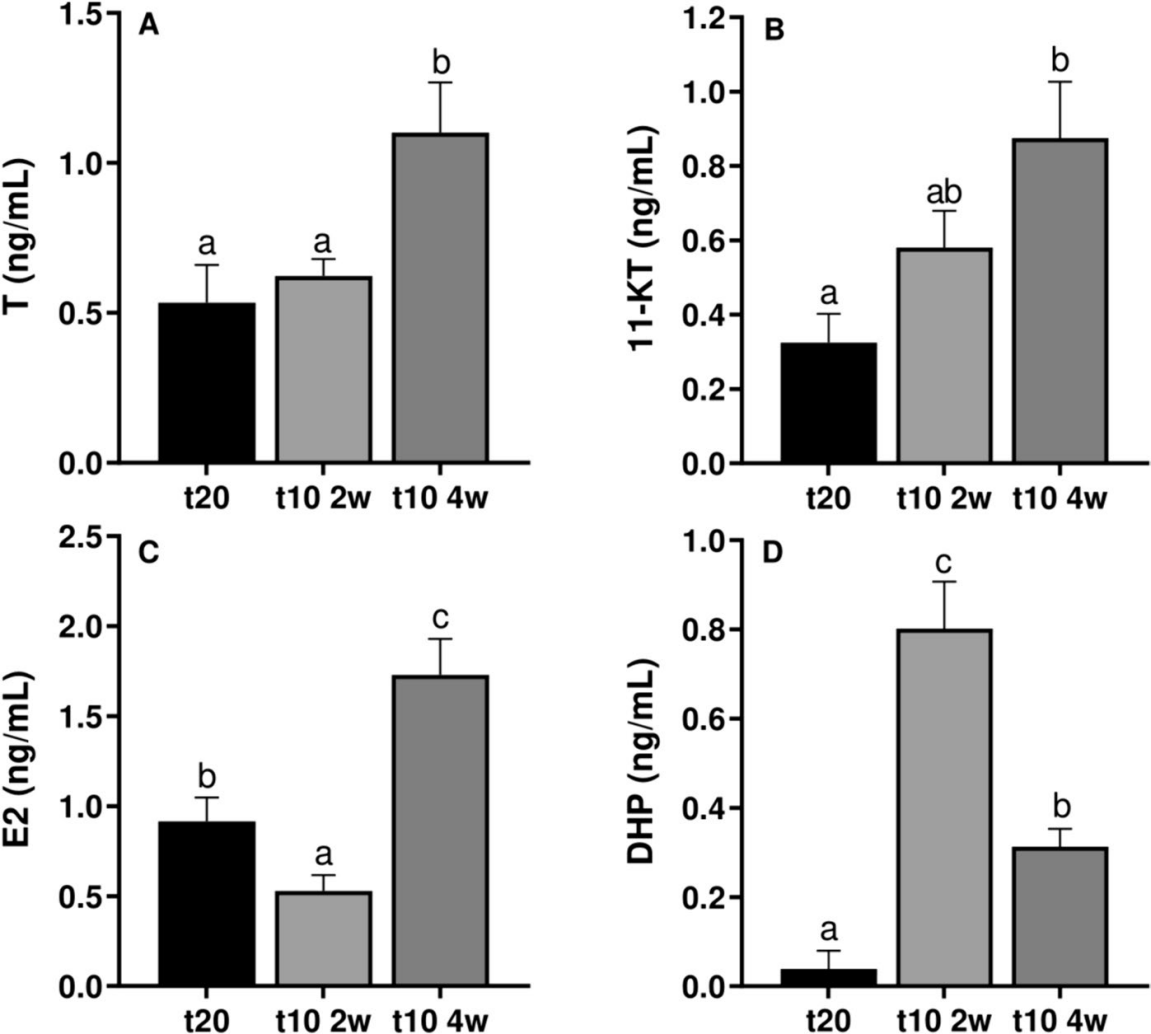
Steroid levels. T (**A**), 11-KT (**B**), E2 (**C**) and DHP (**D**) levels from control (t20), 2-week (t10 2w), and 4-week (t10 4w) pre-treated groups (n = 8 per treatment and sampling). Data are expressed as mean ± SEM and different letters indicate significant differences (one-way ANOVA followed by *pos-hoc* SNK test, *p* < 0.05) between the experimental groups

During blood steroid analyses, males pre-treated for 4 weeks showed higher concentrations of T plasma levels than the control group and those pre-treated for 2 weeks (*p* < 0.01) (Fig. 4A). For 11KT, male eels pre-treated for 4 weeks registered significantly higher plasma levels than the control group, while those pre-treated for 2 weeks showed intermediate values (*p* < 0.01) (Fig. 4B). Concerning the E2, the highest levels were observed in males pre-treated for 4 weeks, followed by the control and the males pre-treated for 2 weeks, which exhibited the lowest E2 levels (*p* < 0.001) (Fig. 4C). DHP levels in males pre-treated for 2 weeks were significantly higher in comparison with those pre-treated for 4 weeks and the control group, which recorded the lowest levels (*p* < 0.001) (Fig. 4D).

### Sperm production

**Fig. 5:**
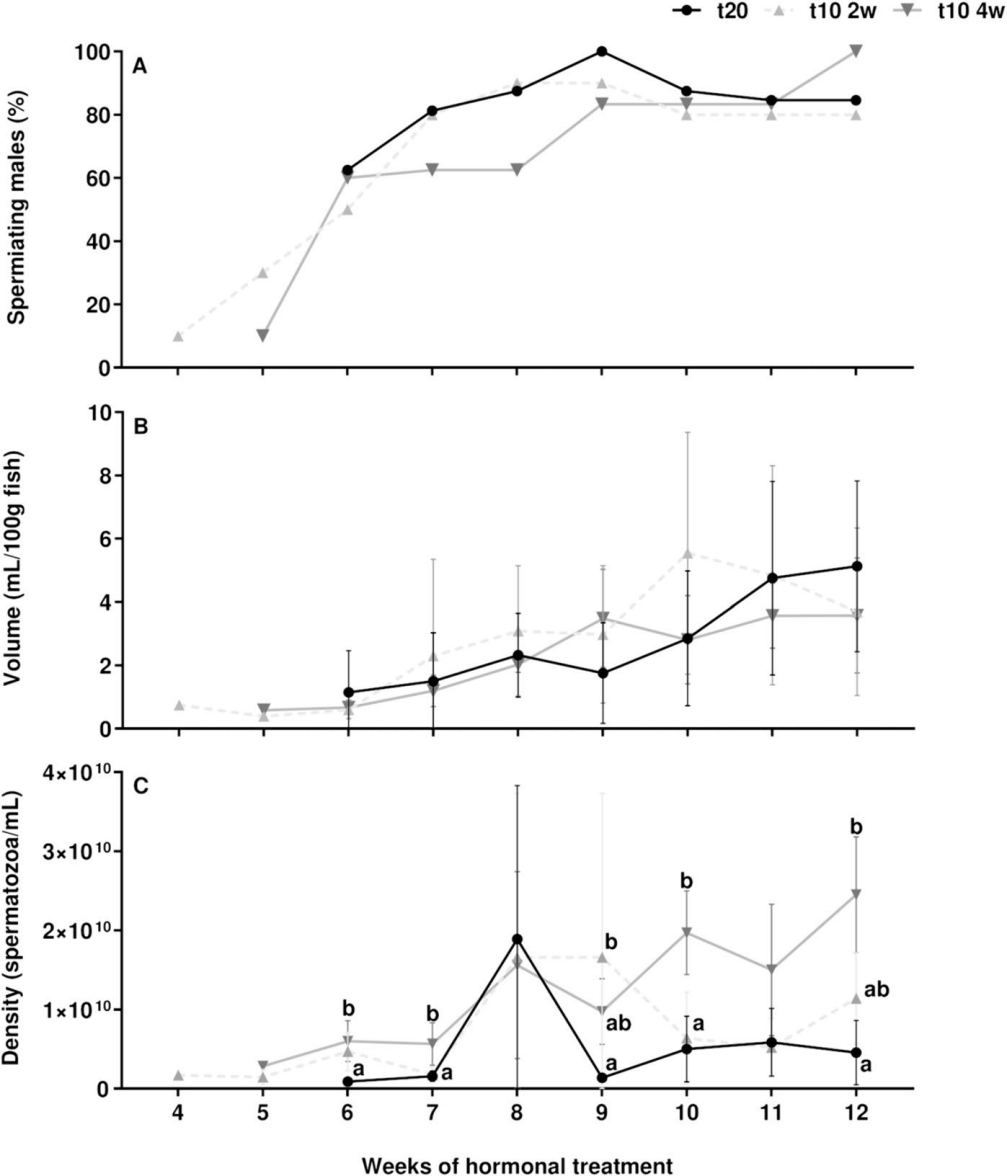
Time course of sperm production. (**A**) Percentage of spermiating males, (**B**) sperm volume (mL/100 g fish) and (**C**) density (spermatozoa/mL) values between control (t20), 2-week (t10 2w), and 4-week (t10 4w) pre-treated groups. Data are expressed as mean ± SEM and different letters indicate significant differences (one-way ANOVA followed by *pos-hoc* SNK test or Kruskal–Wallis, *p* < 0.05) between the experimental groups

The sperm production parameters are shown in Fig. 5. There was a pre-treatment-dependent response as both pre-treated groups responded earlier to hCG_rec_ treatment (Fig. 5A). The control males started to produce sperm after 6 weeks of hormonal treatment, while at least part of the males from the 2 and 4-week pre-treated groups spermiated after 4 and 5 weeks, respectively. In the following weeks, the control and 2-week pre-treated males generated higher percentages of spermiating males than the 4-week pre-treated males. All the groups showed similar percentages of spermiating males between weeks 9 and 11. At week 12 the 4-week pre-treatment showed the highest percentage of spermiating males.

Regarding sperm volume, there was an increasing trend from the beginning to the end in all treatments, although sperm volumes showed no significant differences between the different groups (Fig. 5B). The 2-week pre-treated group reached its peak spermiation volume (5.54 ± 3.82 mL/100 g fish) by week 10. In contrast, both the control and the 4-week pre-treated groups achieved their maximum sperm volume values (5.13 ± 2.69 and 3.58 ± 1.81 mL/100 g fish, respectively) by week 12.

Concerning the sperm density (Fig. 5C), both pre-treated groups showed higher values than the control group in week 6 (*p* < 0.001) and 7 (*p* < 0.001) of the hormonal treatment. At week 8, all groups registered similar density values, but one-week later 2-week pre-treated males showed significantly higher sperm density than control eels (*p* < 0.05). At week 10, this pattern changed, and 4-week pre-treated males reached significantly higher density (*p* < 0.001). At week 11, no differences were reached but at the last week of hormonal treatment, males pre-treated for 4-weeks reached the maximum value (2.45 × 10^10^ ± 7.32 × 10^9^ spermatozoa/mL), contrasting with control males.

### Sperm motility and kinetic parameters

**Fig. 6:**
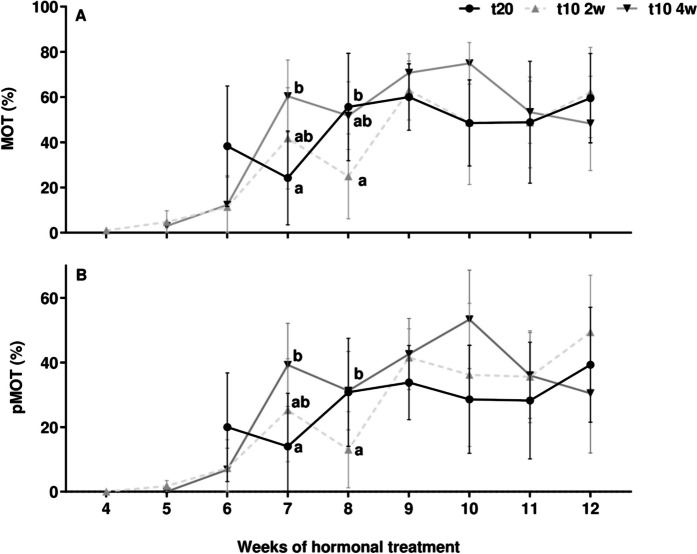
Time course of sperm motility parameters. Percentage of (A) total motile (MOT, %) and (B) progressive motile (pMOT, %) cells between control (t20), 2-week (t10 2w), and 4-week (t10 4w) pre-treated groups. Data are expressed as mean ± SEM and different letters indicate significant differences (one-way ANOVA followed by *pos-hoc* SNK test or Kruskal–Wallis, *p* < 0.05) between each experimental group and at the week of hormonal treatment

Both the control and pre-treated males showed a gradual increase in MOT and pMOT sperm throughout the standard hormonal treatment (Fig. 6A and B, respectively). At week 7, the 4-week pre-treated group exhibited significantly higher MOT (*p* < 0.01) and pMOT (*p* < 0.05) in comparison to the control group, but not compared to the 2-week pre-treated group. At week 8, MOT (*p* < 0.01) and pMOT (*p* < 0.05) from 2-week pre-treated group were lower than the remaining groups. However, the following weeks and until the end of the hormonal treatment, the control and both pre-treated groups yielded high values of MOT and pMOT sperm motility, without significant differences.

**Fig. 7:**
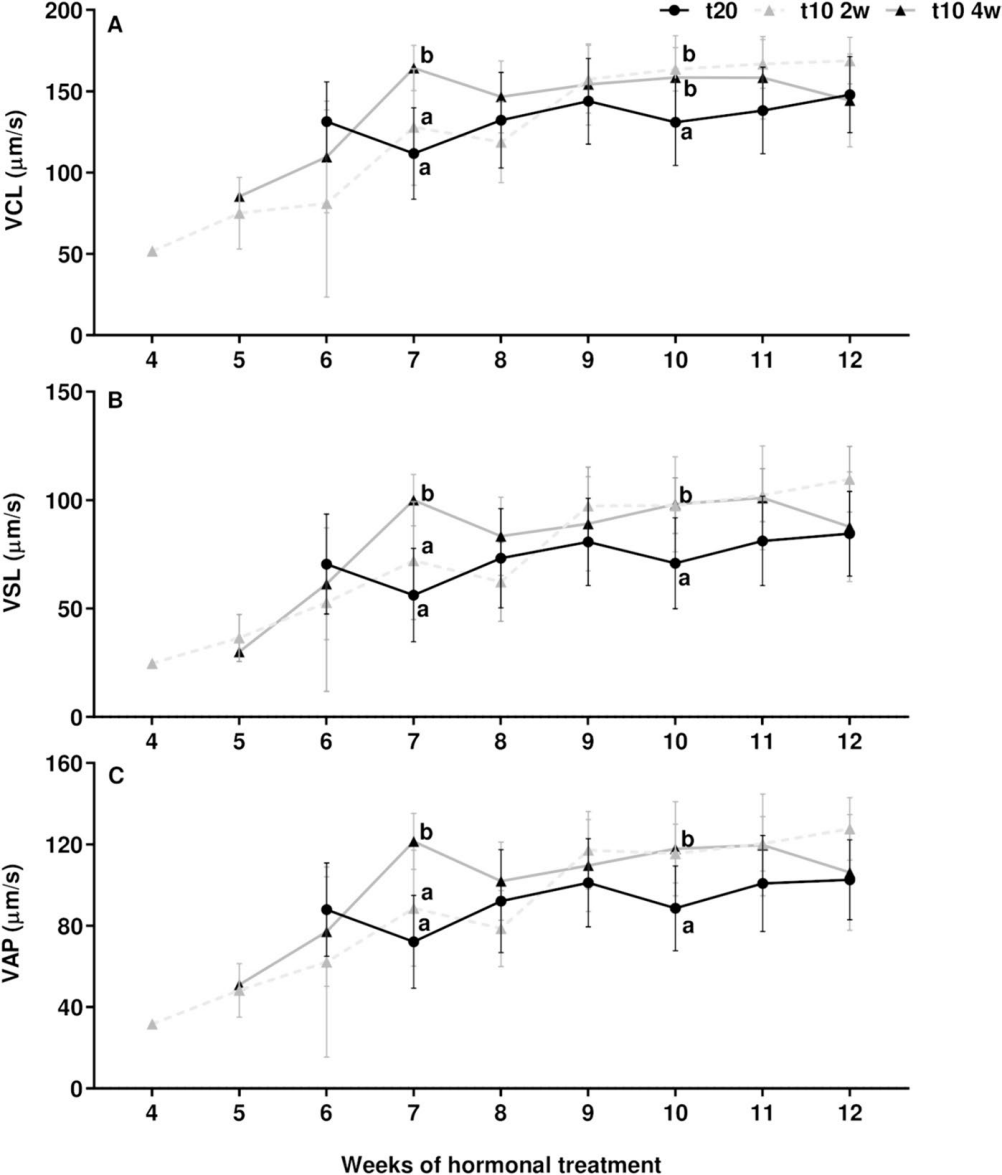
Time course of sperm velocity parameters. Velocity according to (**A**) curvilinear path (VCL, µm/s), (**B**) straight-line path (VSL, µm/s) and (**C**) average path (VAP, µm/s) between control (t20), 2-week (t10 2w), and 4-week (t10 4w) pre-treated groups. Data are expressed as mean ± SEM and different letters indicate significant differences (one-way ANOVA followed by *pos-hoc* SNK test or Kruskal–Wallis, *p* < 0.05) between each experimental group and at the week of hormonal treatment

Regarding sperm kinetic parameters, there was a general increase of the values, with just punctual significant differences between groups at some weeks (Fig. 7). The 4-week pre-treated males showed significantly higher VCL (*p* < 0.01), VSL (*p* < 0.01) and VAP (*p* < 0.01) values by week 7 (Fig. 7A, B and C, respectively). Both pre-treated groups showed significantly higher VCL (*p* < 0.01), VSL (*p* < 0.01) and VAP (*p* < 0.01) values than the control group at week 10.

### Hormonal treatment profitability

The control and both pre-treated groups required an equal investment per male (Table 1; 1.17 €/100 g of fish for 12 weeks treatment). Concerning the total volume of sperm produced per male, the 2-week treated group provided a higher volume (1.67 mL/100 g) in comparison with the control and the 4-week pre-treated groups (1.33 and 1.05 mL/100 g of fish). However, the total volume of high-quality sperm (> 70%) was higher in the 2 and 4-week pre-treated males (0.54 and 0.61 mL/100 g of fish, respectively). Therefore, the hormonal treatment had a better profitability when was preceded by a low temperature pre-treatment, as it was possible to obtain 1 mL of the highest quality sperm for a lower price (2.16 and 1.93 €/mL, in 2- and 4-week pre-treated groups respectively). In contrast, control males without the cold seawater pre-treatment had the worst profitability, with 1 mL of good quality sperm costing 6.46 €.

**Table 1:** Hormonal treatment profitability estimation in relation to hormone injections investment and production of high-quality sperm between control (t20), 2-week (t10 2w) and 4-week (t10 4w) pre-treated groups. High-quality sperm volume refers to quantity of produced sperm with motility equal or higher than 70% of motile cells by control (n = 14), 2-week (n = 14) and 4-week (n = 15) pre-treated spermiating males during the standard treatment duration

	t20	t10 2w	t20 4w
Total sperm volume/male (mL/100 g of fish)	1.33	1.67	1.05
High-quality sperm volume/male (mL/100 g of fish) ^a^	0.18	0.54	0.61
High-quality sperm price ^b^	6.46	2.16	1.93

^a^High-quality sperm volume (total volume of sperm with motility equal or higher than 70% of motile cells)

^b^Total investment (€)/ Total high-quality sperm volume (mL)

## Discussion

The present study shows that low temperature pre-treatment stimulates early stages of sexual maturation in the European eel. Eels maintained in 10 °C seawater for 4 weeks registered an increase of the gonadosomatic index, together with a slight increase pattern (not statistically significant) in the eye and hepatosomatic indexes. These morphological changes are considered part of the silvering process during reproductive migration, preparing them for the conditions encountered during their reproductive journey (Dufour et al. 2003; Sébert et al. 2008). In our study, the impact of the duration of low temperature seawater pre-treatment on the eel maturation process, particularly in gonad development, was evident. Rozenfeld et al. (2019) study on male European eels maintained in cold seawater without hormonal treatment for only two weeks showed no significant differences in the biometric parameter indexes. Moreover, the same study did not register the gonadosomatic index after this period due to testis weight being excessively low (Rozenfeld et al. 2019). In contrast, our study suggests that a longer exposure (i.e., 4 weeks) to low temperature seawater (without hormonal treatment) was necessary to stimulate an increase in gonad weight. The effect of low temperature on gonad development has been confirmed in the present study as it has been previously observed in the Atlantic cod (*Gadus morhua,* Davie et al. 2007; Hansen 2001), pollack (*Pollachius pollachius*, Suquet et al. 2005), and in the Eurasian perch (*Perca fluviatilis,* Migaud et al. 2002).

The histological results from our study suggested that cold seawater treatment induces spermatogonial differentiation in the testes of the European eel. The gonadosomatic index results suggest that a 4-week cold seawater pre-treatment might have increased the weight of gonads as a result of the proliferation effect. Moreover, SPGAdiff and SPGB cell proportions were higher in both cold seawater pre-treated groups, indicating that low-temperature seawater stimulated spermatogonial differentiation into more advanced stages. This did not occur in the control group as SPGAdiff was the predominant cell type but the proportions of SPGAund and SPGB were similar. In teleosts, the spermatogonia proliferation and differentiation processes are known to be regulated during spermatogenesis by different steroid hormones (Miura et al. 2003; Schulz et al. 2010). Although E2 levels in male teleosts are typically low (Schulz et al. 2010), Miura et al. (1999) demonstrated in the Japanese eel that E2 stimulates spermatogonial stem renewal proliferation through receptors, as it was later confirmed in the European eel testis by Morini et al. (2017a). The second stage of spermatogenesis is modulated by androgens, in particular 11-KT, which acts as a factor in the initiation of spermatogonial proliferation, as was suggested by Miura et al. (2011) for the Japanese eel and later evidenced in the European eel (Peñaranda et al. 2016). In our study, the 4-week pre-treated group exhibited higher levels of E2 and androgens (T and 11-KT), which might have stimulated the inferred gonadal development, increasing the gonadosomatic index together with SPGAdiff cells proliferation. Our conclusion coincides with Rozenfeld et al. (2019) findings in European eels, which maintained males at low temperature without hormonal treatment and also registered an increase of T and 11-KT levels, that consequently stimulated SPGAdiff proliferation and differentiation. Moreover, E2 and 11-KT levels increased in female Japanese eels after water temperature decrease (Sudo et al. 2011). Together, these studies and our results suggest that cold seawater pre-treatment promotes an increase in E2 and both androgen levels, which induces an increase in the weight of gonads as a consequence of the SPGAdiff cell proliferation and differentiation processes.

Our results with cold seawater pre-treatment suggest that low temperature modulates the DHP levels in male eels. In teleosts, DHP is mostly known as an important maturation-inducing steroid (MIS), which has a role in the late spermatogenesis processes (Scott et al. 2010). However, previous studies in European eel testis suggested that DHP also participates in early spermatogenesis stages via some specifically expressed membrane and nuclear receptors (Morini et al. 2017b). In hCG-treated European eel, Peñaranda et al. (2016) reported an increase in both E2 and androgen levels in males maintained at 10 °C, while DHP levels remained low until temperature increase. In the present study, males pre-treated for 2 weeks exhibited higher levels of DHP compared to those found in pre-treated males for 4 weeks, suggesting an early proliferative effect of DHP in response to cold seawater. The lower DHP levels in 4-week pre-treated males suggest that the proliferation effect had already occurred in the testis. Our results support that DHP has an important role in early spermatogenesis for eel as suggested previously by Miura et al. (2006) in the Japanese eel.

Collectively, the present study indicates that cold seawater has a time-dependent effect on E2, androgen and DHP levels, probably by regulating the synthesis of steroidogenic enzymes responsible for steroid pathway shifts during the early stages of spermatogenesis.

The effect of low temperature pre-treatment combined with standard hormonal treatment (hCG_rec_ weekly injections at 20 °C) was investigated by evaluating sperm quantity and quality, complementing the previous studies that considered only the initial stages of the spermatogenesis (Rozenfeld et al. 2019). In the present study, males from the pre-treated groups started sperm production earlier, and volumes from 2-week treated males reached 4 mL/100 g fish, which is consistent with previous results obtained in hCG-induced European eels kept at high temperatures (Gallego et al. 2012). No significant differences in sperm volume were observed between groups, but males pre-treated with low temperature displayed higher sperm density values (except week 8 and 11). Moreover, the 4-week pre-treated males reached a higher density value at week 12, suggesting that this group would maintain the sperm production for a longer period. In terms of sperm kinetic parameters, males pre-treated with cold seawater showed higher sperm motility and velocity values in some treatment weeks (week 7, 8 and 10). In the European sea bass (*Dicentrarchus labrax*), several increases in plasma androgen levels precede MIS (such as DHP) production, and these shifts in gonadal steroidogenesis led to consecutive spawning and waves of sperm maturation (Asturiano et al. 2002). Moreover, Miura et al. (2006) suggested that DHP is an important MIS in teleosts, having a crucial role in sperm maturation and sperm movement. In our study, males kept at low temperature for longer pre-treatments exhibited significantly elevated androgen plasma levels prior to be transferred to 20 °C and receiving hCG_rec_ injections. These results suggest that cold seawater pre-treatment prior to standard hormonal treatment may have resulted in a rapid steroid conversion of androgens towards the synthesis of DHP, that acts as a sperm maturation factor in late spermatogenesis, leading to early spermiation and higher sperm quality.

An optimal hormonal treatment should provide abundant and high-quality sperm samples in terms of both volume and density, maintaining good sperm motility and velocity for a long spermiation period (Herranz-Jusdado et al. 2019a, b). Similarly, it is economically crucial to decrease the expenses associated with hormonal therapies to make treatments more affordable and efficient (Mylonas et al. 2017). Our study estimated the economic efficiency of the cold seawater pre-treatments by considering the investment of hormone injections and its productivity in terms of the total volume and quality of sperm. It is worth noting that in our study, the hormone injection purchase is the only fixed cost and the remaining costs (in especial electricity required for water cooling at different temperatures) were challenging to assess and, therefore, were not considered. Our primary objective was to evaluate the cost of the hormonal treatment and determine whether pre-treated eels showed increased sensitivity to standard treatment and increased sperm quantity and quality. Considering the price of high-quality sperm, both groups with thermal pre-treatment generated the best results, suggesting that low temperature pre-treatment improves the performance yielded by the control group. In this sense, the investment required for maturing males submitted to control and low temperature pre-treatments coincided with previous studies in the European eel (Herranz-Jusdado et al. 2019a, b; Gallego et al. 2012). Therefore, with thermal pre-treatment, males produced larger amounts of good quality sperm, reducing the cost efficiency of the hormonal treatment cost.

## Conclusions

The present study describes the effects of low temperature pre-treatments from early spermatogenesis to spermiation in the European eel. The changes in biometric parameters and spermatogonia differentiation were enhanced by the pre-treatment with seawater at 10 °C, probably as result of increased steroid levels. After standard treatment (weekly hCGrec injections at 20 °C), pre-treated males responded earlier to the hormonal injections, and sperm motility and sperm kinetic parameters during some weeks were higher than those found in eels without pre-treatment. In addition, the low temperature pre-treatments demonstrated their economic effectiveness, increasing the production of high-quality sperm in European eel.

## Data Availability

No datasets were generated or analysed during the current study.
